# Carotid Atherosclerosis and T-Cell Imbalance in Women with Rheumatoid Arthritis: A Cross-Sectional Study of Intima–Media Thickness, Anti-CCP Antibodies and CD4/CD8 Ratio

**DOI:** 10.3390/jcm15135054

**Published:** 2026-06-29

**Authors:** Elena Deseatnicova, Alesea Nistor, Ana Tigulea, Maia Grosu, Stela Dodu, Eugeniu Russu, Lucia Andries, Liliana Groppa

**Affiliations:** 1 Discipline of Rheumatology and Nephrology, Department of Internal Medicine, Nicolae Testemitanu State Medical and Pharmaceutical University, MD-2004 Chisinau, Moldova; alesea.nistor@usmf.md (A.N.); maia.grosu@usmf.md (M.G.); eugeniu.russu@usmf.md (E.R.); liliana.groppa@usmf.md (L.G.); 2 Timofei Moșneaga Republican Clinical Hospital, MD-2025 Chisinau, Moldova; tigulea.ana@gmail.com; 3 Institute of Cardiology, MD-2025 Chisinau, Moldova; steladodu@gmail.com; 4 Laboratory of Immunology, Nicolae Testemitanu State Medical and Pharmaceutical University, MD-2004 Chisinau, Moldova; lucia.andries@usmf.md

**Keywords:** rheumatoid arthritis, carotid intima–media thickness, atherosclerosis, cardiovascular risk, CD4/CD8 ratio, anti-CCP antibodies, immunosenescence

## Abstract

**Background:** Rheumatoid arthritis (RA) is associated with increased cardiovascular risk beyond traditional risk factors. Carotid intima–media thickness (IMT) is a marker of subclinical atherosclerosis and may be influenced by chronic inflammation in RA. **Objectives:** This study aimed to compare carotid IMT between women with RA and age-matched controls, and to investigate the associations between IMT and RA-related factors, including disease duration, menopausal status, anti-cyclic citrullinated peptide (anti-CCP) antibodies and the CD4/CD8 T-cell ratio. **Methods:** This cross-sectional study included 59 women with RA and 55 age-matched controls. All RA patients received methotrexate and had no recent biologic DMARD exposure. Carotid IMT was measured by B-mode ultrasonography. Clinical, laboratory, and immunologic parameters, including anti-CCP antibodies and CD4/CD8 ratio, were analyzed. **Results:** Mean carotid IMT was significantly higher in RA patients than in controls (0.85 ± 0.09 vs. 0.63 ± 0.08 mm, *p* < 0.0001). Within the RA group, IMT correlated positively with disease duration (r = 0.48, *p* < 0.001), years since menopause (ρ = 0.44, *p* = 0.001), anti-CCP titers (r = 0.31, *p* = 0.018) and CD4/CD8 ratio (r = 0.42, *p* < 0.001), and inversely with CD8 T-cell counts (r = −0.34, *p* = 0.009). In multivariable analysis, RA duration and CD4/CD8 ratio remained independently associated with IMT after adjustment for age, lipids and blood pressure. **Conclusions:** Women with RA had greater carotid IMT than age-matched controls. Longer disease duration and a higher CD4/CD8 ratio were independently associated with IMT, supporting a link between cumulative disease burden, T-cell imbalance and subclinical carotid atherosclerosis in RA. These findings support routine cardiovascular risk assessment and consideration of vascular imaging in high-risk RA subgroups.

## 1. Introduction

Rheumatoid arthritis (RA) is a chronic systemic autoimmune disease that confers a substantially elevated burden of cardiovascular disease (CVD). Recent systematic reviews indicate that the prevalence of CVD in RA is more than two-fold higher than in the general population, underscoring its major contribution to RA-related morbidity and mortality [1,2]. Large cohort studies and meta-analyses consistently demonstrate that RA is associated with a 1.5–2-fold increase in incident cardiovascular events, even after adjustment for traditional risk factors such as age, hypertension and dyslipidemia [2,3]. A landmark meta-analysis including over 40,000 RA patients further quantified this excess risk, reporting a 48% increase in overall cardiovascular events and a 68% higher risk of myocardial infarction compared with non-RA controls [4]. Importantly, accumulating evidence suggests that the cardiovascular hazard associated with RA is comparable in magnitude to that of type 2 diabetes mellitus, which has led major clinical societies to classify RA as an independent cardiovascular risk-enhancing condition [5].

The pathogenesis of atherosclerosis in RA is complex and multifactorial, involving a dynamic interaction between traditional cardiovascular risk factors and RA-specific inflammatory and immunologic mechanisms. Chronic systemic inflammation, mediated by cytokines such as tumour necrosis factor-α (TNF-α) and interleukin-6 (IL-6), contributes to endothelial dysfunction, oxidative stress, prothrombotic activation and accelerated plaque formation [6,7,8]. Clinically, these processes manifest as a robust association between RA disease activity and adverse cardiovascular outcomes, with patients exhibiting persistently high disease activity demonstrating greater carotid plaque progression and higher rates of cardiovascular events than those achieving remission [9,10,11]. Sustained elevation of inflammatory markers, including C-reactive protein (CRP), has also been directly linked to endothelial damage and increased carotid intima–media thickness (IMT) in RA [12].

Beyond cytokine-driven inflammation, RA-specific autoantibodies and cellular immune abnormalities may directly contribute to vascular injury. Anti-citrullinated protein antibodies (anti-CCPs), present in approximately 70–80% of RA patients, exhibit potential atherogenic effects. Citrullinated proteins have been identified within human atherosclerotic plaques, and anti-CCPs may bind these epitopes to form immune complexes in the arterial wall, amplifying local inflammation and promoting atherogenesis [13,14,15]. Several studies report that anti-CCP-positive RA patients exhibit more extensive subclinical atherosclerosis and a higher risk of ischemic heart disease than seronegative individuals, although the strength of the association varies across cohorts [16,17].

RA is also characterised by profound T-lymphocyte dysregulation, including expansions of pro-inflammatory, senescent CD4^+^CD28^−^ and CD8^+^CD28^−^ T-cell subsets, often linked to chronic antigenic stimulation and latent cytomegalovirus infection [18,19]. These immunosenescent T-cell populations secrete high levels of interferon-γ and cytotoxic mediators and have been associated with endothelial dysfunction, coronary artery calcification and increased carotid IMT in RA [20]. Dysregulation of CD8^+^ regulatory/cytotoxic T cells may further impair vascular immune homeostasis, facilitating chronic arterial inflammation.

Carotid intima–media thickness measured by high-resolution B-mode ultrasound is a validated, non-invasive surrogate marker of subclinical atherosclerosis and an independent predictor of future cardiovascular events in the general population. Large cohort studies and meta-analyses demonstrate that both baseline IMT and IMT progression correlate with incident myocardial infarction and stroke and have been used as surrogate outcomes in cardiovascular clinical trials [21,22,23]. Accordingly, carotid ultrasound has become an increasingly valuable tool for assessing preclinical vascular disease in RA [24]. Meta-analyses consistently show that RA patients display thicker carotid IMT and a higher prevalence of plaques than age- and sex-matched controls, supporting the concept of accelerated atherosclerosis [25,26,27,28]. However, the magnitude of IMT differences varies substantially among studies and the specific contributions of RA-related factors, such as disease duration, autoantibody status, systemic inflammatory burden and immune-cell phenotypes, remain incompletely understood [25,26,27,28,29].

Many earlier cohorts were heterogeneous in terms of sex distribution, age, disease duration, and exposure to biologic DMARDs, making it difficult to isolate the effects of hormones, menopause and immunosenescence. Moreover, detailed immunologic profiling (including T-cell subsets and CD4/CD8 ratio) has rarely been integrated with vascular imaging and relatively few studies have focused specifically on postmenopausal women, who may face synergistic cardiometabolic and immunologic risks [27,29,30].

Given these uncertainties, professional societies emphasize aggressive cardiovascular risk assessment in RA. The European League Against Rheumatism (EULAR) recommends regular CVD risk evaluation with a 1.5 multiplication factor applied to traditional risk calculators, while the American Heart Association identifies chronic inflammatory diseases, including RA, as cardiovascular “risk enhancers” [31,32]. Carotid ultrasound is highlighted as an informative modality to refine risk stratification in selected high-risk individuals [21,22].

In this context, we conducted a focused study of female RA patients to characterize subclinical carotid atherosclerosis and its immunologic and clinical correlates. We hypothesized that women with RA would have increased carotid IMT compared with matched controls and that IMT would correlate with RA-specific factors, including disease duration, cumulative inflammatory burden, anti-CCP levels and immunologic markers such as the CD4/CD8 ratio. By restricting our cohort to patients receiving a uniform treatment regimen (methotrexate) and excluding recent biological exposure, we aimed to minimize treatment-related confounding and isolate disease-related vascular risk. We also specifically examined menopausal status and duration, given the potential interplay between estrogen deficiency, inflammation and immunosenescence. Our overarching goal was to integrate ultrasonographic, clinical and immunologic data to identify determinants of vascular risk in RA and inform strategies for early detection and prevention.

Study objective: This study aimed to assess carotid atherosclerosis in women with rheumatoid arthritis and explore its associations with disease duration, inflammatory burden, immune profile (T-cell subsets and anti-CCP) and menopausal status, using a uniformly treated cohort to identify key predictors of cardiovascular risk.

## 2. Materials and Methods

### 2.1. Study Design and Participants

We conducted a cross-sectional observational study at a single academic rheumatology centre. The study population consisted of two groups: (1) women with rheumatoid arthritis (RA) and (2) age-matched women without RA serving as controls. All RA patients were recruited from ambulatory rheumatology clinics and fulfilled the 2010 American College of Rheumatology/European Alliance of Associations for Rheumatology (ACR/EULAR) classification criteria for RA. The analysis was restricted to women because the source RA population was predominantly female and sex-related differences in cardiovascular risk, immune-cell distribution, hormonal status and carotid IMT could introduce additional heterogeneity in a modest-sized cohort. This design also enabled focused assessment of menopausal status and years since menopause as potential contributors to subclinical atherosclerosis in RA.

RA disease duration was defined as the number of years from the date of verified RA diagnosis, documented in the medical record and based on fulfillment of the 2010 ACR/EULAR classification criteria, to the date of study assessment. Disease duration was not calculated from the first onset of symptoms in order to minimize recall bias.

Inclusion criteria for the RA group were: female sex; age 40–75 years; an established RA diagnosis; and disease duration between 0 and 25 years. To ensure immunologic homogeneity, only patients receiving conventional therapy with methotrexate (with or without low-dose glucocorticoids) were eligible. To minimise the influence of immunomodulatory biologics on T-cell parameters, individuals who had received any biologic disease-modifying antirheumatic drug (bDMARD) within the preceding six months were excluded. Exclusion criteria for RA patients included comorbidities that could independently influence atherosclerosis or immune function: uncontrolled chronic conditions (such as decompensated liver cirrhosis, active viral hepatitis or stage 4–5 chronic kidney disease), diabetes mellitus, prior clinical atherosclerotic cardiovascular disease (coronary or cerebrovascular events), active malignancy or recent cancer therapy, malabsorption syndromes, chronic pulmonary disease (e.g., severe asthma) and other autoimmune or rheumatic disorders. Patients with active infections or pregnancy at the time of assessment were also excluded. The control group consisted of women without RA, frequency-matched to RA patients within ±5 years of age. Controls were recruited from non-biological relatives of patients, community volunteers and hospital staff. They were required to have no history of inflammatory joint disease, autoimmune disorders, diabetes mellitus, clinical CVD, or other major illnesses, and were not permitted to use immunosuppressive or anti-inflammatory medications.

All participants provided written informed consent prior to enrollment. The study protocol was approved by the institutional ethics committee, and all procedures were conducted in accordance with the Declaration of Helsinki. Data collection occurred between 2022 and 2024. For RA participants, clinical information was obtained through medical record review, structured interviews, and standardized physical examination, including assessment of disease activity.

### 2.2. Clinical and Laboratory Assessments

We collected demographic data (age), menopausal status and years since menopause (for postmenopausal participants), as well as traditional cardiovascular risk factors including smoking history, hypertension, hyperlipidaemia and body mass index (BMI). In the RA group, additional disease-specific variables were obtained, including disease duration (years since diagnosis), current disease activity assessed using the Disease Activity Score in 28 joints with C-reactive protein (DAS28-CRP), and current antirheumatic therapy (weekly methotrexate dose and daily glucocorticoid dose, if applicable). Because of the modest sample size and unequal distribution of patients across DAS28-CRP categories, disease activity was analyzed primarily as a continuous variable rather than by formal subgroup comparison. Exploratory descriptive comparisons between disease activity categories were performed where appropriate, but these were interpreted cautiously due to limited statistical power. Rheumatoid factor (RF) and anti-cyclic citrullinated peptide (anti-CCP) antibodies were measured using standard immunonephelometry and enzyme-linked immunosorbent assay (ELISA), respectively, with positivity defined according to the manufacturers’ cut-off values.

All participants underwent peripheral venous blood sampling after an overnight fast. Laboratory analyses included a lipid profile (total cholesterol, HDL cholesterol, LDL cholesterol and triglycerides), high-sensitivity C-reactive protein (hsCRP), and a complete blood count.

**Figure 1:**
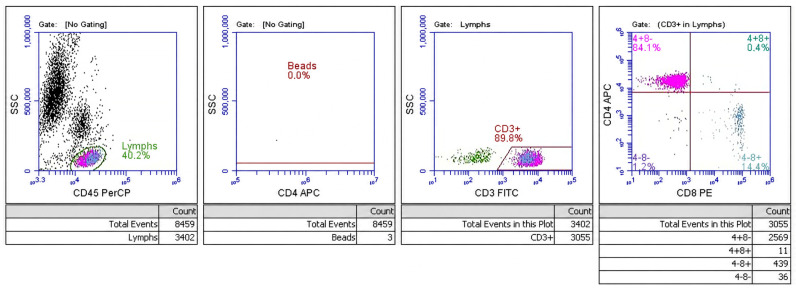
Representative flow-cytometry gating strategy for CD4^+^ and CD8^+^ T-cell identification in a patient with rheumatoid arthritis. Lymphocytes were first identified based on CD45 expression and side-scatter characteristics, representing 40.2% of total acquired events. Within the lymphocyte gate, CD3^+^ T lymphocytes were selected, accounting for 89.8% of lymphocyte events. CD4^+^ and CD8^+^ T-cell subsets were then quantified within the gated CD3^+^ lymphocyte population using CD4 APC and CD8 PE expression. In this representative rheumatoid arthritis sample, CD4^+^CD8^−^ helper T cells represented 84.1% of CD3^+^ T cells, while CD4^−^CD8^+^ cytotoxic T cells represented 14.4%. Double-positive CD4^+^CD8^+^ cells and double-negative CD4^−^CD8^−^ cells accounted for 0.4% and 1.2%, respectively. This gating sequence was used to determine CD4^+^ and CD8^+^ T-cell proportions and to calculate the CD4/CD8 ratio.

Immunologic profiling was performed using flow cytometry on K_3_ EDTA-anticoagulated blood to determine T-lymphocyte subsets, which was processed within 4 h after collection. T-lymphocyte subsets were analyzed by multiparameter flow cytometry using fluorochrome-conjugated monoclonal antibodies directed against CD3, CD4, and CD8 (Figure 1).

**Figure 2:**
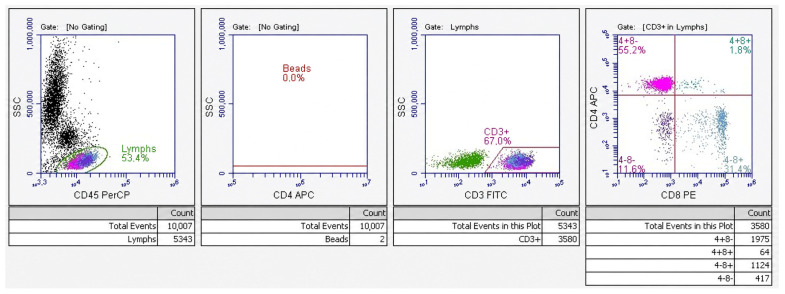
Representative flow-cytometry gating strategy for CD4^+^ and CD8^+^ T-cell identification in a control participant. Lymphocytes were first identified based on CD45 PerCP expression and side-scatter characteristics, representing 53.4% of total acquired events. Within the lymphocyte gate, CD3^+^ T lymphocytes were selected and accounted for 67.0% of lymphocyte events. CD4^+^ and CD8^+^ T-cell subsets were then quantified within the gated CD3^+^ lymphocyte population using CD4 APC and CD8 PE expression. In this representative control sample, CD4^+^CD8^−^ helper T cells represented 55.2% of CD3^+^ T cells, while CD4^−^CD8^+^ cytotoxic T cells represented 31.4%. Double-positive CD4^+^CD8^+^ cells and double-negative CD4^−^CD8^−^ cells accounted for 1.8% and 11.6%, respectively. This example illustrates a more balanced CD4/CD8 distribution compared with the representative rheumatoid arthritis sample.

The antibody cocktail comprised FITC-conjugated anti-CD3 (clone SK7; mouse IgG1κ; 2.3 µg/mL), PE-conjugated anti-CD8 (clone SK1; mouse IgG1κ; 1.75 µg/mL), PerCP-conjugated anti-CD45 (clone 2D1; mouse IgG1κ; 7.50 µg/mL), and APC-conjugated anti-CD4 (clone SK3; mouse IgG1κ; 0.92 µg/mL) (BD Multitest™ CD3/CD8/CD45/CD4; BD Biosciences, Becton, Dickinson and Company, Milpitas, CA, USA). Appropriate isotype controls and fluorescence-minus-one controls were used in accordance with the laboratory protocol (Figure 2).

Whole blood aliquots were incubated with the antibody cocktail for 30 min at room temperature in the dark. Red blood cells were lysed using BD FACS^TM^ Lysing Solution, (BD Biosciences, San Jose, CA, USA), and the remaining leukocytes were washed and resuspended in phosphate-buffered saline prior to acquisition. Data were acquired on a BD FACS Via^TM^, flow cytometer (Becton, Dickinson and Company [BD Biosciences], San Jose, CA, USA) and at least 25,000 lymphocyte events were collected for each sample. Instrument calibration and compensation were performed according to the manufacturer’s recommendations.

The gating strategy was as follows. First, lymphocytes were identified on the basis of forward scatter and side scatter properties. Doublets and debris were excluded where applicable. Within the lymphocyte gate, CD3^+^ cells were identified as total T lymphocytes. CD4^+^ helper T cells and CD8^+^ cytotoxic T cells were then quantified within the CD3^+^ T-cell population and expressed as percentages of total lymphocytes and CD3^+^ T cells. Absolute CD4^+^ and CD8^+^ T-cell counts were calculated using a dual-platform approach, combining the percentage of each T-cell subset obtained by flow cytometry with the absolute lymphocyte count measured by the automated hematology analyzer. The CD4/CD8 ratio was calculated by dividing the absolute CD4^+^ T-cell count by the absolute CD8^+^ T-cell count.

Representative flow-cytometry gating plots for CD4^+^ and CD8^+^ T-cell identification are shown in Figure 1 and Figure 2. Lymphocytes were first identified using forward- and side-scatter properties, followed by gating on CD3^+^ T cells and subsequent quantification of CD4^+^ helper and CD8^+^ cytotoxic T-cell subsets.

Additional immunologic markers (e.g., immunoglobulin isotypes) were measured but were not included in the present analysis, which focused specifically on T-cell distribution.

### 2.3. Carotid Ultrasound and IMT Measurement

Carotid intima–media thickness (IMT) was assessed using high-resolution B-mode ultrasonography with a linear-array transducer (7–12 MHz). All examinations were performed by a certified sonographer who was blinded to participants’ clinical characteristics. Both the left and right common carotid arteries (CCA) were evaluated following a standardised acquisition protocol. IMT was measured at the far wall of the distal CCA, at least 5 mm proximal to the carotid bulb, in a segment free of visible plaque. To optimise measurement reproducibility, the insonation angle was adjusted to obtain a longitudinal view with clearly distinguishable intima–media interfaces, ideally at an angle close to 90°.

For each side, three end-diastolic frames were captured and IMT was quantified as the distance between the lumen–intima and media–adventitia interfaces using semi-automated edge-detection software. The mean IMT value for each participant was calculated as the average of left and right measurements. Carotid plaque was defined as a focal structure encroaching into the arterial lumen by ≥50% of the surrounding IMT or with an absolute thickness > 1.5 mm. Plaques were documented when present, although plaque characteristics were not included in the current analysis, which focused on IMT as a continuous measure of subclinical atherosclerosis.

Quality assurance included regular calibration of ultrasound equipment with phantom imaging and intra-observer reproducibility testing, yielding an intraclass correlation coefficient of 0.92 for repeated IMT measurements.

### 2.4. Statistical Analyses

Statistical analyses were performed using SPSS version 25.0 (IBM Corp., Armonk, NY, USA) and GraphPad Prism (version 9, GraphPad Software, Boston, MA, USA). Continuous variables were inspected for normality using histograms and the Shapiro–Wilk test and are presented as mean ± standard deviation (SD) or median (interquartile range), as appropriate. Categorical variables are presented as counts and percentages. Between-group comparisons (RA vs. controls) were performed using the Student-t test or Mann–Whitney U test for continuous variables and χ^2^ or Fisher’s exact test for categorical variables.

Within the RA group, associations between carotid IMT and clinical or immunologic variables (disease duration, DAS28-CRP, years since menopause, anti-CCP titers, CD4 and CD8 counts, CD4/CD8 ratio, LDL cholesterol, systolic blood pressure) were examined using Pearson or Spearman correlation coefficients, according to distribution. To identify independent predictors of IMT in RA, we constructed multivariable linear regression models including variables with *p* < 0.10 in univariable analysis, while checking for multicollinearity (variance inflation factor < 2.5). A separate model including both RA patients and controls assessed the effect of RA status (binary predictor) on IMT after adjustment for age, blood pressure, LDL cholesterol and smoking status. Model results are reported as regression coefficients (β) with 95% confidence intervals (CI) and *p* values. A two-sided *p* value < 0.05 was considered statistically significant. Given the exploratory nature of the analyses, no formal correction for multiple comparisons was applied, but effect sizes and confidence intervals are provided to aid interpretation.

## 3. Results

### 3.1. Participant Characteristics

Fifty-nine women with RA and 55 age-matched controls were included. Mean age was similar in the two groups (54.8 ± 6.3 vs. 53.9 ± 6.5 years, *p* = 0.48), and 91.5% (54 out of 59 study group) and 92.7% (51 out of 55 in control group) were postmenopausal (Table 1). RA patients had a slightly longer postmenopausal duration than controls (5.52 ± 3.4 vs. 4.45 ± 2.6 years, *p* < 0.05), while the prevalence of smoking, hypertension and mean body mass index were comparable. By design, none of the participants had diabetes or documented clinical CVD (Table 1).

RA patients had established disease (mean duration 8.3 ± 6.7 years) and predominantly moderate disease activity (mean DAS28-CRP 4.19 ± 0.8). Patients were unevenly distributed across disease activity categories: 22% had high disease activity, 61% had moderate disease activity, and 17% had low disease activity or remission. Mean carotid IMT was numerically higher among patients with high disease activity than among those with low disease activity/remission; however, the association between DAS28-CRP as a continuous variable and IMT did not reach statistical significance. Therefore, disease activity was not used as the primary grouping variable for intergroup comparison.

All the patients in the study received methotrexate (mean dose 17.5 mg/week) and 80% were on a low dose of glucocorticosteroids (GCS), but total lymphocyte counts were largely within the normal range at the time of assessment. In total 90.9% of patients had history of GCS use. No patient had received biologic DMARDs or JAK inhibitors within 6 months. Seropositivity was frequent (78% RF-positive, 73% anti-CCP-positive). High-sensitivity CRP levels were significantly elevated in RA vs. controls (8.1 ± 5.6 vs. 2.0 ± 1.8 mg/L, *p* < 0.001).

The lipid profile demonstrated a distinct, though modest, pattern among patients with RA. Compared with controls, RA patients exhibited lower mean levels of total cholesterol and LDL cholesterol (mean LDL: 114 vs. 123 mg/dL, *p* = 0.04; total cholesterol: 192 vs. 202 mg/dL, *p* = 0.07). HDL cholesterol levels were also lower in the RA group (50 vs. 55 mg/dL), although this difference did not reach conventional statistical significance (*p* = 0.08). Collectively, these findings are consistent with the well-described “lipid paradox” in RA, whereby systemic inflammation is associated with suppressed circulating cholesterol levels despite an increased risk of cardiovascular disease.

The total cholesterol-to-HDL cholesterol ratio, a marker of atherogenic risk, was numerically higher in RA patients than in controls (4.0 vs. 3.8), although this difference was not statistically significant (*p* = 0.20). Triglyceride concentrations were comparable between the two groups. Statin use was similar among RA patients and controls (20% vs. 18%, respectively; *p* = 0.81) and exclusion of individuals receiving statin therapy did not materially alter the observed lipid differences.

Blood pressure levels were well controlled and did not differ meaningfully between groups (median 130/80 mmHg in RA patients vs. 128/78 mmHg in controls). Overall, aside from trends suggestive of an inflammation-modified lipid profile in RA, the two groups were broadly comparable with respect to traditional cardiovascular risk factors, supporting the interpretation that RA-specific disease mechanisms may represent the primary drivers of differential CV risk in this population.

Patients with RA demonstrated differences in T-cell subset distributions compared with controls. The mean absolute CD4^+^ T-cell count was higher in the RA group (820 ± 210 cells/µL) than in controls (760 ± 180 cells/µL), whereas the mean absolute CD8^+^ T-cell count was lower in RA patients (320 ± 140 cells/µL) compared with controls (440 ± 150 cells/µL). Accordingly, the CD4:CD8 ratio was significantly increased in RA patients (2.72 ± 0.84) relative to controls (1.78 ± 0.50; *p* < 0.001). A CD4:CD8 ratio exceeding the upper limit observed in the control group was present in 81% of RA patients.

Between-group differences were statistically significant for the CD8^+^ T-cell count and the CD4:CD8 ratio (*p* < 0.05 for both), whereas the difference in CD4^+^ T-cell count did not reach statistical significance (*p* = 0.06). No significant differences were observed in total lymphocyte count or neutrophil count between groups. Serum immunoglobulin levels (IgG, IgM, and IgA) were assessed; although higher IgG and IgA levels were observed in RA patients compared with controls, these measures were not significantly associated with carotid intima–media thickness and are therefore not presented in detail.

### 3.2. Carotid Intima-Media Thickness in RA vs. Controls

All participants underwent bilateral carotid ultrasonography with successful acquisition of IMT measurements. Mean carotid IMT was significantly higher in patients with rheumatoid arthritis (RA) compared with controls (0.85 ± 0.09 mm vs. 0.63 ± 0.08 mm, *p* < 0.0001).

**Figure 3:**
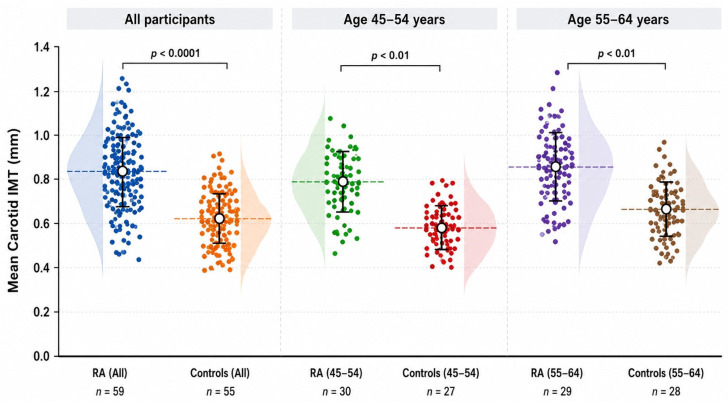
Distribution of carotid IMT in women with RA and age-matched controls. Jittered scatter plots and violin distributions illustrate individual IMT values across the overall cohort and age-specific subgroups (45–54 and 55–64 years). Open circles and error bars represent mean ± SD. RA patients exhibited significantly greater IMT than controls in all age categories, indicating a higher burden of subclinical atherosclerosis.

The difference in IMT between RA patients and controls was observed across all age strata. Among women aged 45–54 years, mean IMT was 0.80 mm in the RA group versus 0.58 mm in controls, while among those aged 55–64 years, corresponding values were 0.87 mm and 0.66 mm, respectively (all *p* < 0.01). The distribution of IMT values showed minimal overlap between groups, with the majority of RA patients exhibiting IMT values above the 75th percentile of the control distribution (Figure 3).

Using a threshold of IMT ≥ 0.75 mm, 49 of 59 RA patients (83%) met or exceeded this value, compared with 5 of 55 controls (9%). Carotid plaque, defined as a focal lesion with thickness > 1.5 mm, was detected in 22 RA patients (37%) and in 8 controls (15%) (*p* = 0.01).

Within the RA group, carotid IMT was positively correlated with disease duration (Pearson r = 0.48, *p* < 0.001). Patients with RA duration greater than 10 years had higher mean IMT values than those with shorter disease duration (0.92 ± 0.08 mm vs. 0.81 ± 0.09 mm, *p* < 0.01). IMT also correlated with years since menopause (Spearman ρ = 0.44, *p* = 0.001). RA patients with ≥5 years since menopause had higher IMT than those within 5 years of menopause (0.89 ± 0.08 mm vs. 0.80 ± 0.09 mm, *p* = 0.004). No significant correlation between menopause duration and IMT was observed in the control group.

We next examined whether carotid IMT in RA patients correlated with their disease-related variables. RA duration showed a strong positive correlation with IMT: Pearson r = +0.48 (*p* < 0.001). Thus, each additional year of RA was associated with incremental carotid thickening. Patients with RA duration > 10 years had notably high IMT (0.92 ± 0.08 mm on average), approaching levels associated with clinical coronary disease in other studies. This relationship remained significant after controlling for age, suggesting that prolonged exposure to the RA disease process accelerates vascular aging beyond chronological aging. We also found that IMT correlated moderately with cumulative inflammatory burden. We did not have formal area-under-curve inflammation measures, but CRP at the time of study and DAS28-CRP as a snapshot of disease activity both showed trends: IMT was higher in the subset of RA patients with high disease activity (mean 0.90 mm) compared to those in remission/low activity (0.78 mm), and the correlation between DAS28 as a continuous variable and IMT was positive (r = +0.20) though not statistically significant (*p* = 0.13). These results hint that current disease activity contributes, but the influence of long-term inflammation (captured by disease duration or perhaps serologic status) is more pronounced. Indeed, seropositivity emerged as an important correlate: patients who were anti-CCP positive had a higher mean IMT than those who were anti-CCP negative (0.88 vs. 0.77 mm, *p* = 0.01). Moreover, anti-CCP antibody levels showed a linear correlation with IMT (r = +0.31, *p* = 0.018). In a simple stratification, IMT averaged 0.90 mm in the highest anti-CCP titer quartile versus 0.80 mm in the lowest quartile. Rheumatoid factor positivity showed a similar trend but was less discriminating (likely because nearly all RF+ patients were also anti-CCP+).

Menopause-related factors also appeared relevant. We observed a positive correlation between years since menopause and IMT within the RA group (Spearman ρ = +0.44, *p* = 0.001). Postmenopausal RA patients with ≥5 years since menopause had significantly greater IMT than those within 5 years of menopause (0.89 vs. 0.80 mm, *p* = 0.004), even after adjusting for age (which was collinear with menopause duration). This suggests that the compounding effect of prolonged estrogen deficiency in RA might exacerbate atherosclerosis. Notably, menopause duration did not correlate with IMT in the control group (*p* = 0.45), hinting that the effect is more pronounced in the inflammatory context of RA.

**Figure 4:**
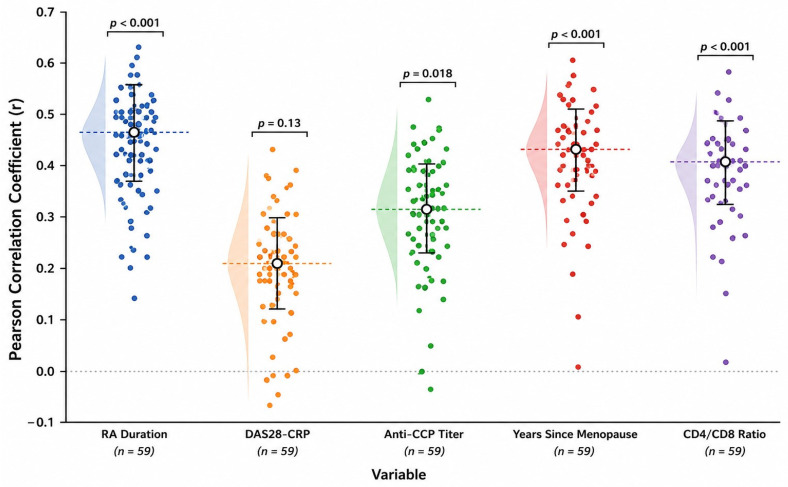
Scatter plots showing the associations between carotid IMT and RA-related variables. Linear regression lines with 95% confidence intervals are displayed. Significant positive correlations were observed for RA duration, anti-CCP antibody titers, years since menopause, and CD4/CD8 ratio, whereas DAS28-CRP showed no significant association.

To better understand independent contributors to IMT, we performed a multivariable linear regression among RA patients. Variables entered included age, RA duration, DAS28-CRP, anti-CCP positivity, LDL level, systolic blood pressure, and CD4/CD8 ratio. In the final model, RA duration (β = 0.45, *p* < 0.001) and CD4/CD8 ratio (β = 0.28, *p* = 0.012) emerged as significant independent predictors of carotid IMT (Figure 4), while age (β = 0.20, *p* = 0.07) showed a strong trend.

LDL and blood pressure were not significant predictors in this cohort with relatively homogeneous traditional risk factor profiles. Notably, when anti-CCP positivity was replaced by actual titer, the titer was an independent predictor (*p* = 0.03), but if using just seropositive vs. negative, it did not reach significance (likely due to limited power and the high seropositive rate). These results suggest that disease chronicity and immunologic characteristics of RA are associated with greater subclinical atherosclerosis, although causality cannot be inferred from the present cross-sectional data, over and above “classic” risk factors.

In the combined analysis of RA patients and controls, RA status was associated with an average IMT increase of +0.22 mm (95% confidence interval 0.18–0.26 mm) after adjusting for age, blood pressure, LDL, and smoking (*p* < 0.0001). This illustrates that middle-aged women with RA have carotid IMT values equivalent to women roughly 20 years older in the general population. Such an effect size aligns with epidemiologic data that RA confers a cardiovascular risk comparable to adding 5–10 years of age or to having diabetes.

### 3.3. Immunologic Correlates of Atherosclerosis in RA

Alterations in T-lymphocyte subsets were associated with carotid intima–media thickness in patients with RA. The CD4/CD8 T-cell ratio showed a significant positive correlation with IMT (r = 0.42, *p* < 0.001). Consistently, patients in the highest tertile of the CD4/CD8 ratio (>3.0) had greater IMT than those in the lowest tertile (<2.2) (0.93 vs. 0.78 mm, *p* = 0.0004). In multivariable analysis adjusted for age and RA duration, the CD4/CD8 ratio remained significantly associated with IMT.

Analysis of the individual T-cell subsets showed that CD8^+^ T-cell count was inversely correlated with IMT (r = −0.34, *p* = 0.009), whereas CD4^+^ T-cell count showed a weaker, non-significant positive correlation (r = 0.18, *p* = 0.17). Nearly all patients were receiving low-dose prednisone, and total lymphocyte counts were largely within the normal range.

Among the other immune and inflammatory markers examined, total white blood cell count showed a modest positive correlation with IMT (r = 0.25, *p* = 0.051). IL-6 was available in a subset of patients and showed a positive trend with IMT (Spearman’s ρ ≈ 0.30). Anti-CCP-positive patients also had higher CD4/CD8 ratios on average than anti-CCP-negative patients. Overall, among the immunologic variables assessed, the CD4/CD8 ratio showed the strongest association with IMT.

## 4. Discussion

In this cross-sectional study of middle-aged women with rheumatoid arthritis, we found substantially greater carotid intima–media thickness in RA than in age-matched controls, together with a high prevalence of pathological IMT values and carotid plaques. Within the RA group, carotid IMT was most strongly associated with disease duration and with the CD4/CD8 T-cell ratio, while anti-CCP titers and years since menopause also showed significant relationships. Taken together, these findings support the concept that subclinical atherosclerosis in RA reflects the combined influence of chronic inflammatory exposure, serologic disease severity, hormonal status and adaptive immune dysregulation.

The novelty of the present study lies not in demonstrating increased carotid IMT in RA alone, which has been reported previously, but in integrating vascular ultrasound with RA-specific clinical, hormonal, serological, and immunologic parameters in a relatively homogeneous female cohort. In particular, the combined assessment of disease duration, menopausal status, anti-CCP titers, CD4/CD8 ratio, and carotid IMT provides additional insight into a potentially high-risk phenotype of women with RA. Clinically, these findings suggest that cardiovascular risk assessment in RA should not rely only on traditional risk factors, but may be strengthened by incorporating disease-related and immune markers together with carotid ultrasound in selected patients. Our results are broadly consistent with the literature showing increased subclinical atherosclerosis in RA and with prospective studies indicating that persistent moderate or high disease activity promotes plaque progression [10]. Recent evidence also underscores important heterogeneity across RA cohorts. In the 8-year ELSA-Brasil case–cohort analysis, RA was not independently associated with baseline carotid IMT and progression, or carotid plaque prevalence after adjustment for sociodemographic and cardiovascular risk factors [33]. In contrast, the population-based Paracelsus 10,000 study found that RA remained independently associated with carotid plaque after adjustment for SCORE2, metabolic syndrome, age, sex, and hs-CRP [34]. Prospective data further suggest that this variability is biologically plausible: persistent moderate or high disease activity increases the risk of subclinical atherosclerosis progression, whereas cohorts maintained in sustained low disease activity or remission may show attenuated or no excess progression relative to controls. Moreover, the vascular effect of disease activity may be most apparent in patients with lower baseline conventional cardiovascular risk [10,35]. Taken together, these findings suggest that cardiovascular risk in RA is not uniform but phenotype-specific, likely shaped by cumulative inflammatory burden, effectiveness of disease control, therapeutic context and background cardiometabolic risk. In this context, our cohort—predominantly postmenopausal, seropositive women with moderate disease activity and no recent biologic exposure may represent a higher-risk subgroup rather than the average contemporary RA population.

A particularly novel aspect of our study was the association between T-cell imbalance and carotid IMT. The CD4/CD8 ratio remained independently associated with IMT. This relationship appeared to be driven more by lower circulating CD8^+^ T-cell counts than by absolute CD4^+^ expansion. This observation fits with emerging concepts of premature immune aging in RA [36]. Recent work has emphasised that RA is characterised by features of immunosenescence, especially within T-cell compartments, and prior RA studies have linked CMV seropositivity and expansion of CD4^+^CD28null and CD8^+^CD28null cells with greater carotid IMT [36,37]. Although the CD4/CD8 ratio was associated with carotid IMT in our cohort, this marker provides only a broad measure of T-cell imbalance. We did not assess CMV serostatus or senescent T-cell subsets, such as CD4^+^CD28null and CD8^+^CD28null cells, which have been implicated in immune aging and vascular risk in RA. Therefore, our findings should not be interpreted as direct evidence of CMV-driven immunosenescence. Rather, the elevated CD4/CD8 ratio may reflect an accessible surrogate of adaptive immune dysregulation that requires further characterization in future studies. Nevertheless, the persistence of this association after adjustment for age and RA duration suggests that immune phenotype may contribute incremental vascular information beyond standard clinical variables.

The association between anti-CCP titers and IMT in our study is also noteworthy. Previous work has shown that RF isotypes and anti-CCPs may be associated with incident cardiovascular events in RA and experimental data support biologic plausibility for a vascular role of citrullinated antigens [38]. However, more recent evidence suggests that systemic inflammation may be more closely related to cardiovascular mortality than anti-CCP status alone [39]. Our data therefore support interpreting anti-CCPs as a marker of a more severe or immunologically active RA phenotype, rather than as definitive evidence of direct antibody-mediated vascular injury.

From a clinical perspective, our findings support intensified cardiovascular surveillance in selected RA subgroups. EULAR recommends structured cardiovascular risk assessment in RA, and recent studies continue to show that conventional risk algorithms may fail to identify many patients with carotid plaque [40,41,42,43]. In this setting, carotid ultrasound may be particularly useful in seropositive patients, in those with longer disease duration and in those with evidence of immune dysregulation. Because carotid plaque appears more closely linked to future cardiovascular events than IMT alone, future studies should determine whether immune markers such as the CD4/CD8 ratio are more strongly associated with plaque burden or plaque progression than with IMT.

Our data reinforces the importance of suppressing inflammatory burden over time. Prospective studies indicate that disease activity contributes to plaque development, supporting the concept that vascular protection in RA is closely tied to effective control of systemic inflammation [35,44]. The therapeutic implications should nevertheless be stated cautiously. While some treatments may improve vascular profiles indirectly by reducing inflammation, cardiovascular safety differs across drug classes. The relevant randomized safety signal for JAK inhibition comes from ORAL Surveillance, whereas observational registries have yielded more heterogeneous results [45,46,47]. Accordingly, our findings are best interpreted as supporting careful cardiovascular phenotyping and individualized treatment selection rather than favoring any specific drug class.

This study benefited from a relatively homogeneous female RA cohort on methotrexate-based therapy, age-matched controls, and standardized carotid ultrasound with concurrent T-cell subset assessment. However, modest sample size and single-center, cross-sectional design limited power, subgroup analyses and causal inference. Residual confounding cannot be excluded, and immune characterization was limited to CD4^+^ and CD8^+^ counts without assessment of CMV serostatus, senescent T-cell phenotypes, endothelial function or arterial stiffness. These results therefore require validation in larger longitudinal studies.

## 5. Conclusions

In this cross-sectional study of women with rheumatoid arthritis, carotid intima–media thickness and plaque prevalence were higher than in age-matched controls despite broadly similar conventional cardiovascular risk profiles. Within the RA group, longer disease duration and an elevated CD4/CD8 ratio were independently associated with greater IMT, while anti-CCP titers and years since menopause showed additional univariable associations. These findings demonstrate an association between cumulative disease burden, adaptive immune dysregulation and subclinical carotid atherosclerosis in RA, but do not establish a causal relationship. Prospective studies incorporating plaque-based outcomes and deeper immune phenotyping are needed to determine whether the CD4/CD8 ratio can improve cardiovascular risk stratification in RA.

## 6. Practical Implications and Future Perspectives

The findings of this study have practical relevance for cardiovascular risk assessment in patients with rheumatoid arthritis. The combination of routinely available clinical data, inflammatory markers, anti-CCP antibodies, basic T-cell subset analysis and carotid ultrasound may help physicians identify patients with a higher burden of subclinical vascular disease. In clinical practice, particular attention may be warranted in RA patients with longer disease duration, persistent inflammatory activity, anti-CCP positivity, postmenopausal status and evidence of immune imbalance, such as an increased CD4/CD8 ratio.

For specialists, these findings support a more integrated approach to cardiovascular risk assessment in RA, combining traditional cardiovascular risk factors with RA-specific and immunologic indicators. For a broader clinical audience, the main message is that cardiovascular risk in RA may not be fully explained by cholesterol levels, blood pressure or smoking alone. Chronic inflammation and immune dysregulation may also be associated with early vascular changes and should be considered when evaluating the patient’s overall cardiovascular condition.

In the future, artificial intelligence (AI) and clinical decision-support systems may help integrate these different types of information, including disease duration, disease activity, lipid profile, inflammatory markers, autoantibody status, immune-cell parameters and carotid ultrasound findings. Such tools could assist clinicians in identifying high-risk patients more quickly and linking laboratory and imaging abnormalities to the patient’s clinical condition. However, AI-based risk prediction was not evaluated in the present study, and future longitudinal studies are required to develop and validate such models before they can be recommended for routine clinical use.

## Figures and Tables

**Table 1:** The characteristics of participants.

Parameter	RA Group (Mean ± SD or %)	Control Group (Mean ± SD or %)	*p*-Value
Age (years)	54.8 ± 6.3	53.9 ± 6.5	0.48
Postmenopausal duration (years)	5.52 ± 3.4	4.45 ± 2.6	<0.05
Smokers (%)	25%	22%	0.72
Hypertension (%)	30%	30%	—
BMI (kg/m^2^)	26.8	26.1	0.55
RA duration (years)	8.3 ± 6.7	—	—
DAS28-CRP (mean)	4.19 ± 0.8	—	—
DAS28 > 5.1 (%)	22%	—	—
DAS28 3.2–5.1 (%)	61%	—	—
DAS28 < 3.2 (%)	17%	—	—
RF positive (%)	78%	—	—
Anti-CCP positive (%)	73%	—	—
Anti-CCP titer (U/mL)	156 ± 80	—	—
hsCRP (mg/L)	8.1 ± 5.6	2.0 ± 1.8	<0.001
Total cholesterol (mg/dL)	192	202	0.07
LDL (mg/dL)	114	123	0.04
HDL (mg/dL)	50	55	0.08
Triglycerides (mg/dL)	—	—	—
TC/HDL ratio	4.0	3.8	0.2
Statin use (%)	20%	18%	0.81
CD4^+^ T cells (cells/µL)	820 ± 210	760 ± 180	0.06
CD8^+^ T cells (cells/µL)	320 ± 140	440 ± 150	<0.05
CD4/CD8 ratio	2.72 ± 0.84	1.78 ± 0.50	<0.001

## Data Availability

The datasets generated and/or analyzed during the current study are not publicly available due to participant confidentiality and ethical restrictions established by the approved informed-consent framework. Only aggregated data supporting the findings are presented in this article.
